# Study protocol: improving cognition in people with progressive multiple sclerosis: a multi-arm, randomized, blinded, sham-controlled trial of cognitive rehabilitation and aerobic exercise (COGEx)

**DOI:** 10.1186/s12883-020-01772-7

**Published:** 2020-05-22

**Authors:** Anthony Feinstein, Maria Pia Amato, Giampaolo Brichetto, Jeremy Chataway, Nancy Chiaravalloti, Ulrik Dalgas, John DeLuca, Peter Feys, Massimo Filippi, Jennifer Freeman, Cecilia Meza, Matilde Inglese, Robert W. Motl, Maria Assunta Rocca, Brian M. Sandroff, Amber Salter, Gary Cutter, On behalf of the CogEx Research Team

**Affiliations:** 1 Department of Psychiatry, University of Toronto and Sunnybrook Health Sciences Centre, Toronto, ON M5R 3B6 Canada; 2 Department NEUROFARBA, Section Neurosciences, University of Florence, Largo Brambilla 3, 50134 Florence, Italy; 3 IRCCS Fondazione Don Carlo Gnocchi, Florence, Italy; 4 Scientific Research Area, Italian Multiple Sclerosis Foundation (FISM), Via Operai 40, 16149 Genoa, Italy; 5 AISM Rehabilitation Service, Italian Multiple Sclerosis Society (AISM), Via Operai 30, 16149 Genoa, Italy; 6 Queen Square MS Centre, Department of Neuroinflammation, University College London (UCL) Queen Square Institute of Neurology, Faculty of Brain Sciences, UCL, London, UK; 7 Kessler Foundation, East Hanover, NJ USA; 8 Department of Physical Medicine & Rehabilitation, Rutgers New Jersey Medical School, Newark, NJ USA; 9 Section for Sport Science, Department of Public Health, Aarhus University, Dalgas Avenue 4, DK-8000 Aarhus, Denmark; 10 Faculty of Rehabilitation Sciences, Hasselt University, Diepenbeek, Belgium; 11 Neuroimaging Research Unit, Institute of Experimental Neurology, Division of Neuroscience, and Neurology unit, IRCCS, San Raffaele Scientific Institute, Via Olgettina 60, 20132 Milan, Italy; 12 Vita-Salute San Raffaele University, Milan, Italy; 13 Faculty of Health: Medicine, Dentistry and Human Sciences, University of Plymouth, Devon, UK; 14 Department of Neurosciences, Rehabilitation, Ophthalmology, Genetics, Maternal and Child Health (DINOGMI), and Center of Excellence for Biomedical Research, University of Genoa, Genoa, Italy; 15 Department of Physical Therapy, University of Alabama at Birmingham, Birmingham, USA; 16 Division of Biostatistics, Washington University School of Medicine, St. Louis, MO USA; 17 Department of Biostatistics, University of Alabama at Birmingham, Birmingham, USA

**Keywords:** Aerobic exercise, Cognitive training, Progressive multiple sclerosis

## Abstract

**Background:**

Cognitive dysfunction affects up to 70% of people with progressive MS (PMS). It can exert a deleterious effect on activities of daily living, employment and relationships. Preliminary evidence suggests that performance can improve with cognitive rehabilitation (CR) and aerobic exercise (EX), but existing data are predominantly from people with relapsing-remitting MS without cognitive impairment. There is therefore a need to investigate whether this is also the case in people with progressive forms of the disease who have objectively identified cognitive impairment. It is hypothesized that CR and EX are effective treatments for people with PMS who have cognitive impairment, in particular processing speed (PS) deficits, and that a combination of these two treatments is more effective than each individual treatment given alone. We further hypothesize that improvements in PS will be associated with modifications of functional and/or structural plasticity within specific brain networks/regions involved in PS measured with advanced MRI techniques.

**Methods:**

This study is a multisite, randomized, double-blinded, sham controlled clinical trial of CR and aerobic exercise. Three hundred and sixty subjects from 11 sites will be randomly assigned into one of four groups: CR plus aerobic exercise; CR plus sham exercise; CR sham plus aerobic exercise and CR sham plus sham exercise. Subjects will participate in the assigned treatments for 12 weeks, twice a week.

All subjects will have a cognitive and physical assessment at baseline, 12 weeks and 24 weeks. In an embedded sub-study, approximately 30% of subjects will undergo structural and functional MRI to investigate the neural mechanisms underlying the behavioral response. The primary outcome is the Symbol Digit Modalities Test (SDMT) measuring PS. Secondary outcome measures include: indices of verbal and non-verbal memory, depression, walking speed and a dual cognitive-motor task and MRI.

**Discussion:**

The study is being undertaken in 6 countries (11 centres) in multiple languages (English, Italian, Danish, Dutch); with testing material validated and standardized in these languages. The rationale for this approach is to obtain a robustly powered sample size and to demonstrate that these two interventions can be given effectively in multiple countries and in different languages.

**Trial registration:**

The trial was registered on September 20th 2018 at www.clinicaltrials.gov having identifier NCT03679468. Registration was performed before recruitment was initiated.

## Background

A recent comprehensive review of rehabilitation studies in progressive MS (PMS) encompassing balance, weakness, cardiovascular fitness, ataxia, fatigue, bladder dysfunction, spasticity, pain, cognitive deficits, depression and pseudobulbar affect concluded that there was a striking dearth of studies devoted solely to people with secondary progressive MS (SPMS) or primary progressive MS (PPMS) [1]. Furthermore, when including progressive patients alongside patients with relapsing-remitting disease (RRMS), subject numbers has generally been very small while analysis of treatment effects have not accounted for the possible influence of disease course. Finally, in the few studies reporting benefits of treatment for patients with progressive disease, the ecological validity of the results remained uncertain.

The present study focuses on improving cognition in people with PMS for two reasons.

First, up to 70% of people with PMS are impaired in this domain [2] and second, people with MS have themselves identified cognitive dysfunction as a primary area of concern [3]. Furthermore, there are now a number of studies suggesting that cognitive rehabilitation (CR) can result in significant improvements in numerous cognitive domains. A consistent picture is emerging of CR bringing about improvements in learning/memory [4, 5] and processing speed [6, 7]. Of note is that home-based CR programs have also reported significant cognitive gains [8, 9] as have interventions administered in a group setting [10, 11]. Complementing these data are findings suggesting that exercise too can provide physical, cognitive and emotional benefits [12–14].

These results, while encouraging, are also limited by various factors including small sample size, single centre administration and sample composition predominately limited to people with RRMS. Whether people with progressive disease will derive the same benefits from these interventions is therefore not known, although a single study suggests that improvement in memory may be possible with CR [4]. The gaps in our knowledge therefore suggest a number of complementary ways forward. Multisite replication of these preliminary, promising findings is a good place to start. However, as people with progressive MS constantly remind us, time is short. This suggests that an additional effort is required, a bolder approach, one that combines more than one intervention with the aim of producing synergistic effects, an improvement in one area boosting the putative benefits of therapy in another, the overall outcome exceeding the sum of the individual treatments. Such an approach often reflects the clinical reality of PMS where multiple neurological difficulties rather than an isolated problem must be addressed simultaneously. While a powered joint CR and exercise clinical trial has yet to be done in people with MS of any disease course there is tentative evidence from three small studies in people with RRMS that this approach may be more beneficial than either intervention alone [15–17]. Thus, a methodologically rigorous, well-powered study is needed now in PMS specifically in an effort to inform clinical practice. It is only through such a study that we can ensure that final conclusions and recommendations are not limited by sample size, generalizability or methodological concerns.

## Methods/design

The protocol adheres to the Spirit guidelines.

### Aim, design and setting of the study

The study has the following primary aims:

1. To assess whether CR and exercise (EX) in combination have beneficial synergistic effects in the treatment of impaired processing speed in people with PMS.
2. To determine whether CR and EX are individually effective treatments for impaired processing speed in people with PMS.

Secondary aims:

1. To assess the everyday life impact of cognitive and/or physical changes after the different rehabilitation interventions.
2. To assess brain functional and structural substrates of cognitive changes after the different rehabilitation interventions.

**Fig. 1:**
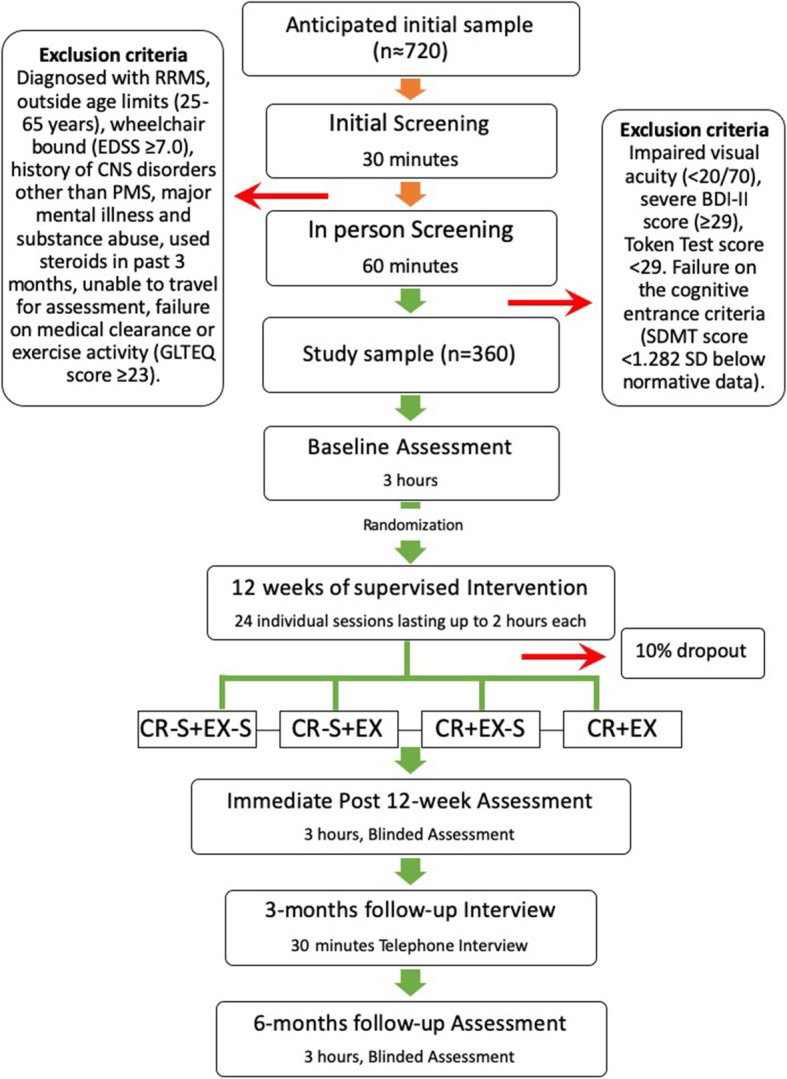
Study flow-chart. RRMS, Relapsing-Remitting Multiple Sclerosis; EDSS, Expanded Disability Status Scale; CNS, Central nervous system; PMS, Progressive Multiple Sclerosis; GLTEQ, Godin Leisure-Time Exercise Questionnaire; BDI-II Beck Depression Inventory-II; SDMT, Symbol Digit Modalities Test; CR-S, cognitive rehabilitation - sham; EX-S, exercise-sham; EX, exercise; CR, cognitive rehabilitation

This study is a multicenter, multi-arm, randomized, double-blinded, sham-controlled trial that includes follow up periods of 12- and 24 weeks (+/− 2 weeks) post randomization. As depicted in Fig. 1, after the baseline assessment each participant will be randomized to one of four arms with different combinations of CR and EX and their respective shams (−S). That is: CR + EX; EX + CR-S; EX-S + CR; EX-S + CR-S.

The study protocol will be carried out at 11 sites in six different countries (Canada (1 site), USA (2 sites), United Kingdom (2 sites), Denmark (1 site), Belgium (1 site) and Italy (4 sites)). Each site has at least one blinded and one unblinded research assistant. The blinded measurement assessor (research assistant) will screen potential participants and perform all baseline and 6-month testing, whereas the 3-month follow up interview will be made by the unblinded research assistant. If a participant meets the inclusion criteria (see later), the unblinded research assistant is responsible for randomizing participant using REDCap (a web-based system) and conducting the interventions for which the participant is assigned. As the study design aims to mask the intent of the intervention given to the participants, the unblinded research assistant will be strictly instructed not to discuss participant allocation and participants will likewise be instructed not to reveal details that can indicate their group allocation to the blinded assessor. The MRI analysis will be undertaken by experienced technicians blinded to group allocation.

### Recruitment and screening of participants

Participants are being recruited via specialized in and out-patient MS clinics, as well as via media advertising. Prior to enrollment, all potential participants will undergo a two-step screening procedure. First, a *pre-screening* examination in person or via telephone will collect basic information. If the participant passes the initial pre-screen, a detailed face-to-face screening for neurological, psychiatric, cognitive, and medical variables will take place at the participating center. Inclusion and exclusion criteria for the two screening steps are summarized in Tables 1 and 2, respectively.

**Table 1:** Inclusion and exclusion criteria for the initial pre-screening step

	**Inclusion criteria**
Diagnosis	A definite diagnosis of PMS determined by neurologist
Age	25–65 years
Ambulation	Not wheelchair dependent (EDSS,< 7.0)
	**Exclusion criteria**
Substance abuse	Use of illicit drugs, PCP, LSD, Stimulants, Amphetamines, Barbiturates, etc. (Cannabis use is accepted).
Neurological history	Relapses in the past 3 months, a history of central nervous system disease other than PMS such as stroke, Parkinson’s disease, traumatic brain injury, etc.
Severe mental illness	Psychotic symptoms, Bipolar Disorder, Schizophrenia.
Medications	Steroid use within the past 3 months.
Transport	Unable or unwilling to travel to the center for testing and training or requiring transportation by ambulance.
Medical contradiction	No medical clearance provided by a physician upon failure of AHA/ACSM Health/Fitness Facility Pre participation Screening Questionnaire.
Current exercise activity	Currently performing enough physical activity for health benefits based a GLTEQ score > 23).

*PCP* Phencyclidine, *LSD* Lysergic acid diethylamide, *AHA* American Heart Association, *ACSM* American College of Sports Medicine, *EDSS* Expanded Disability Status Scale, *GLTEQ* Godin Leisure-Time Exercise Questionnaire, *PMS* Progressive Multiple Sclerosis

**Table 2:** Inclusion and exclusion criteria for the second in-person interview

	**Inclusion Criteria**
SDMT	1.282 SD or more below published normative data (10th percentile).
	**Exclusion criteria**
Visual Acuity	Corrected near vision worse than 20/70 (to see the test materials). Severe nystagmus according to neurologist ratings.
The Beck Depression Inventory	Score ≥ 29 (indicative of severe depression)
Token test	Score ≥ 29 indicative of intact verbal comprehension
MRI sites only	Failing the standard MRI screening form

*MRI* Magnetic Resonance Imaging, *SDMT* Symbol Digit Modalities Test

When participants complete the study they will revert to their neurologist and usual care programs.

### Randomization

Participants will be randomized to a treatment arm using block randomization by site. Block sizes will be blinded to study personnel and principal investigators. Randomization assignment will be conducted in REDCap (a secure web application for building and managing databases) within 24 h after the baseline assessment.

### Interventions

For all interventions, comprehensive manuals were developed and distributed to all participating sites to ensure optimal standardization. This was complemented by face-to-face and distance training at the beginning of the study, and supplemented as necessary during the course of the study. All interventions will be delivered within the hospital/clinic setting or research center under individual supervision, twice per week over a 12 week period. All intervention sessions will start with either CR or CR-S followed by EX or EX-S. In case of holiday, sickness or other unforeseen circumstances the intervention period may be extended by up to two additional weeks, allowing the intervention period to be a maximum of 14 weeks. During the intervention period, compliance to the exercise prescription (attended/planned sessions; actual intensity/target intensity; actual duration/target duration) and cognitive rehabilitation protocols will be monitored by an independent person who will provide regular feedback to sites to optimize the fidelity of the treatment regimens.

Any potential reason for discontinuing will be discussed on individual basis between the principal investigator of the site and the Data Safety Monitoring Board. If a situation occurs where precaution has to be taken in order for the participant to continue in the study, unblinding will be permissible if permitted by the study steering committee.

#### Cognitive rehabilitation (CR)

The CR component comprises the computerized RehaCom program (Pearson’s Clinical Assessment group, Bloomington, MA, USA) that will be performed on non-consecutive days. To address processing speed, the modules “divided attention 1 and 2”, “attention and control”, “sustained attention” and “vigilance 2” will be used. Participants will begin at level 1 and advance the program as dictated by their performance. Each session will be programmed to last 45 min. The RehaCom program has previously been shown to have positive effects on processing speed in persons with RRMS [6, 8, 18].

#### Sham cognitive rehabilitation (CR-S)

The CR-S consists of Internet training [19], beginning with more basic tasks such as learning to use a computer and the internet to search for information, including locating information regarding medications, gardening, getting directions, etc. Each session is programmed to last 45 min and will also take place on non-consecutive days. The control session is designed to match the CR on social and computer contact. Similar training procedures have previously been shown not to impact PS in a normal aging sample [19].

#### Exercise (EX)

The exercise intervention is aerobic and performed on a recumbent stepper (NuStep T5XR, Ann Arbor, MI, USA). All sessions are supervised and performed on an individual basis. Training is performed on non-consecutive days thereby permitting recovery between sessions. The EX intervention consists of twice weekly sessions, one of which is continuous exercise and the other of which is high intensity interval training. It complies with the basic principle of progressive overload. The continuous training ranges from 10 min of exercise at a work rate corresponding with 50–60% of VO_2_peak at week one, progressing to 30 min of exercise at a work rate corresponding with 70–80% of VO_2_peak in week 12. The interval training begins with 5 × 1 min of exercise at a work rate corresponding with 80–90% VO_2_peak followed by 1 min of active breaks with a work rate at 15 watts. At week 12 the protocol will have progressed to 10 × 2 min of exercise at a work rate corresponding with 90% VO_2_peak followed by 2 min of active breaks with a work rate of 15 watts. Protocols built on the same principles have previously improved VO_2_peak in persons with MS [20, 21]. The recumbent stepper represents an acceptable modality of aerobic exercise in people with PMS [22] as has interval training using this device. See Table 3 for further details.

**Table 3:** The progressive aerobic exercise protocol

**Week**	**Session 1 (Continuous training; CT)**	**Session 2 (High Intensity Interval Training; HIIT)**
1	CT (10 min @ WR ~ 50–60% VO2peak)	HIIT (5, 1:1 work:rest intervals; work for 1 min @ WR ~ 80–90% VO2peak, rest for 1 min at 15 W)
2	CT (15 min @ WR ~ 50–60% VO2peak)	HIIT (5, 1:1 work:rest intervals; work for 1.5 min @ WR ~ 80–90% VO2peak, rest for 1.5 min at 15 W)
3	CT (20 min @ WR ~ 50–60% VO2peak)	HIIT (5, 1:1 work:rest intervals; work for 2 min @ WR ~ 80–90% VO2peak, rest for 2 min at 15 W)
4	CT (25 min @ WR ~ 50–60% VO2peak)	HIIT (6, 2:2 work:rest intervals; work for 2 min @ WR ~ 80–90% VO2peak, rest for 2 min at 15 W)
5	CT (30 min @ WR ~ 50–60% VO2peak)	HIIT (7, 2:2 work:rest intervals; work for 2 min @ WR ~ 80–90% VO2peak, rest for 2 min at 15 W)
6	CT (30 min @ WR ~ 50–60% VO2peak)	HIIT (8, 2:2 work:rest intervals; work for 2 min @ WR ~ 80–90% VO2peak, rest for 2 min at 15 W)
7	CT (30 min @ WR ~ 60–70% VO2peak)	HIIT (9, 2:2 work:rest intervals; work for 2 min @ WR ~ 80–90% VO2peak, rest for 2 min at 15 W)
8	CT (30 min @ WR ~ 60–70% VO2peak)	HIIT (10, 2:2 work:rest intervals; work for 2 min @ WR ~ 80–90% VO2peak, rest for 2 min at 15 W)
9	CT (30 min @ WR ~ 65–75% VO2peak)	HIIT (10, 2:2 work:rest intervals; work for 2 min @ WR ~ 90% VO2peak, rest for 2 min at 15 W)
10	CT (30 min @ WR ~ 65–75% VO2peak)	HIIT (10, 2:2 work:rest intervals; work for 2 min @ WR ~ 90% VO2peak, rest for 2 min at 15 W)
11	CT (30 min @ WR ~ 70–80% VO2peak)	HIIT (10, 2:2 work:rest intervals; work for 2 min @ WR ~ 90% VO2peak, rest for 2 min at 15 W)
12	CT (30 min @ WR ~ 70–80% VO2peak)	HIIT (10, 2:2 work:rest intervals; work for 2 min @ WR ~ 90% VO2peak, rest for 2 min at 15 W)

*WR* Work rate, *CT* Continuous training, *HIIT* High intensity interval training

#### Sham exercise (EX-S)

The EX-S does not put strain on the cardiovascular system, so as to avoid a potential aerobic effect, and avoids incorporating progressive resistance strengthening (the use of weights are not permitted) as improvement in aerobic capacity [23] and lower limb muscle strength [24] have been associated with faster cognitive processing speed. Moreover, no focused dual task activities are performed during the training sessions, so as to avoid any potential cognitive training. Hence the EX-S is focusing on balance, co-ordination and stretching, which is a credible sham exercise comparator.

The EX-S sessions have been designed to reflect the EX intervention for time and attention, hence the frequency and duration of sessions, and the manner in which the training times progress throughout the 12 week program mimic that described above. During all EX-S sessions at least one exercise from each of six different categories is performed to allow variation; these are selected on the basis of individual need (exercises summarized in Table 4). To ensure that exercises are at a light intensity, heart rate and rate of perceived exertion (RPE) are monitored throughout the training sessions, after completion of each exercise (i.e. a minimum of six times). Should either the HR or RPE increase above pre-set criteria, an enforced rest is required by the participant to prevent any potential aerobic effect of the exercise. The EX-S protocol builds on a sham intervention that was applied in a previous study in people with MS [25].

**Table 4:** Exercise categories of the sham treatment and the subsequent exercise selection of each category

**Type 1:** **Stretches**	**Type 2:** **Exercises in crook lying**	**Type 3:** **Exercises in side lying**
a) Hamstrings b) Quadriceps c) Hip Flexors d) Hip Abductors e) Bilateral hip abductor f) Ankle plantar-flexors	a) Bridging (two legs/single leg) b) Trunk rotation c) Pelvic tilt d) Unilateral hip abduction/ bilateral hip abduction e) Hip and knee flexion/extension	a) unilateral hip abduction b) unilateral hip abduction/ lateral rotation c) Unilateral knee flexion/extension
**Type 4:** **Exercises in prone**	**Type 5:** **Exercises in unsupported sitting**	**Type 6:** **Exercises in standing**
a) Unilateral hip extension b) Unilateral/bilateral knee flexion c) Bilateral isometric gluteal contractions d) Unilateral/bilateral hip rotation	a) Anterior/posterior pelvic tilt b) Trunk rotation c) Forward trunk flexion d) Unilateral trunk extension (reach out of base of support) e) Unilateral knee extension/flexion f) Unilateral hip abduction g) Bilateral hip abduction	a) Squats (two legs/single leg) b) Step-ups onto low step c) Balancing on one leg (single-leg stance) d) Sideways stepping e) Backwards stepping f) Balancing in step-stance g) Lateral reaching out of base of support

[25]

#### Follow up

To encourage people to continue exercising post supervision the goals of the person with MS will be discussed and taken into account at the beginning of the program, and then reviewed on a further three occasions (every 4 weeks) during the 12 week intervention. This is a practical, and commonly used strategy for positively affecting behavioral change and engagement with rehabilitation programs [26].

### Study status

By March 2020, a total of 135 participants have been randomized into the study. Of these, 40 are now in the intervention phase, 90 have completed the immediate follow up, 63 have completed the 3-month follow up and 37 have completed the 6-month follow up.

### Outcomes and assessments

#### Primary outcome

The primary outcome of this study is the change in processing speed (PS) over the 12 weeks of training, assessed with the Symbol Digit Modalities Test (SDMT). The SDMT is available in several versions, and in the present study, 3 versions will be used in a randomized order to minimize practice effects when repeated [27]. There are several reasons why we chose PS as the primary outcome measure. First, it is well known that PS is the primary cognitive impairment in persons with MS [28] and as a primary cognitive construct, impaired PS itself can lead to problems in higher cognitive functioning such as executive abilities [29]. As such, improving PS may also improve other cognitive areas. Second, there is good preliminary data from smaller studies from multiple laboratories that both CR and exercise improve PS [24, 30–32]. Third, the existing RehaCom literature shows the most consistent and significant effect on PS [18, 33–36]. Fourth, a recent topical review of the SDMT found strong evidence supporting the reliability and validity of the test and recommended a responder definition of SDMT change approximating 4 points or 10% in magnitude [37]. Lastly, after an extensive review of potential cognitive outcome measures by the Multiple Sclerosis Outcome Assessment Consortium, the SDMT was recommended to the Federal regulators as *the* cognitive test of choice to be included in MS clinical trials [38].

#### Secondary outcome

All secondary outcomes will be assessed during the in-person interview or the baseline assessment, at the post 12-week assessment and at the 24-week follow-up assessment.

#### Study assessments

Study assessments are composed of the neuropsychological and exercise assessments, MRI, and the completion of the patient reported outcomes (PROs).

For a detailed overview of all outcomes and the timing of assessment see Table 5.

**Table 5:** Overview of assessments at different test-sessions

**Outcome**	**Initial Screening**	**In person Screening**	**Baseline Assessment**	**12-week intervention**	**Immediate Post 12-week Assessment**	**3-months follow-up Interview**	**6-months follow-up Assessment**
**Cognitive**							
BICAMS*							
**SDMT**		**X**			**X**		**X**
Token Test		X					
BICAMS*							
CVLT			X		X		X
BICAMS*							
BVMT-R			X		X		X
WTAR or NART			X				
**Physical**							
Visual Acuity test		X					
ActiGraph Device		X		X**			
CMI			X		X		X
6MWT			X		X		X
IET			X		X		X
Functional MRI***			X		X		X
**PRO´s**							
Fitness Questionnaire****	X						
GLTEQ	X						
Demographic form		X					
BDI-II		X					
FAMS			X		X		X
EQ-5D-5L			X		X		X
MSIS-29-V2			X		X		X
MSWS-12			X		X		X
PDQ-20			X		X		X
MFIS			X		X		X
HADS			X		X		X
**Customized**							
Phone Interview	X						
Consent form		X					
Medication list		X			X		X
Goal Setting Sheet				X			
Adverse Event form*****				X			
Post Intervention Interview						X	
Post Intervention Survey							X
Serious Adverse Event form*****							X

*Available in three parallel versions;** measured during the week following the intervention; ***only performed at 3 sites; **** AHA/ACSM Health/Fitness Facility Pre participation Screening Questionnaire *****only completed if necessary

Abbreviations: *BICAMS* Brief International Cognitive Assessment for MS, *SDMT* Symbol Digit Modalities Test, *CVLT* California Verbal Learning Test, *BVMT-R* Brief Visuospatial Memory Test-Revised, *WTAR* Wechsler Test of Adult Reading, *NART* National Adult Reading Test, *CMI* Cognitive-Motor Interference, *6MWT* Six Minute Walk Test, *IET* Incremental Exercise Test, *Functional MRI* Functional Magnetic Resonance Imaging, *GLTEQ* Godin Leisure-Time Exercise Questionnaire, *BDI-II* Beck Depression Inventory-II, *FAMS* Functional Assessment of Multiple Sclerosis, *EQ-5D-5L* European Quality of Life-5 Dimensions, *MSIS-29 V2* Multiple Sclerosis Impact Scale-29 Items Version2, *MSWS-12* Multiple Sclerosis Walking Scale 12 Items, *PDQ-20* Perceived Deficits Questionnaire 20 Items, *MFIS* Modified Fatigue Impact Scale, *HADS* Hospital Anxiety and Depression Scale

#### Neuropsychological evaluation

This is conducted in one session to document current levels of cognitive performance. The neuropsychological assessment includes a standard, widely accepted assessment battery for MS, the BICAMS [39]. The BICAMS consists of our primary outcome measure, the SDMT, as well as two other cognitive tests of verbal and visual learning and memory that will be used as secondary outcome measures, namely the California Verbal Learning Test (CVLT) and the Brief Visuospatial Memory Test (BVMT-R). The BICAMS is available in the languages represented within our study sample (English, Italian, French, Dutch and Danish). Language specific normative data are available in all cases [40–43] except in the case for Denmark, where the Dutch norm-data will be applied. Z-scores computed for inclusion criteria used regression-based norms adjusting for linear and non-linear age, sex and total years of education for either the raw or scaled scores from the respective normative data. To provide an assessment of cognitive reserve the Wechsler Test of Adult Reading (WTAR) [44] will be administered at baseline. The WTAR is validated in the participant’s primary language, and an estimated IQ will be computed based on performance. This estimated IQ score will serve as a common metric across all participants for inclusion in analyses. For those countries which do not have WTAR data, the comparable Adult National Reading Test (ANART) [45] will be used.

#### Physical performance

Height and weight will be used to calculate the Body Mass index (BMI). An incremental cardiopulmonary exercise test (CPET) will be conducted to assess peak aerobic capacity and power using the recumbent stepper that is also used for the exercise intervention. The incremental CPET will be undertaken in a standardized manner, and with scripted instructions to the participant. Expired gases will be collected using a 2-way non-rebreathable valve (e.g. Hans Rudolph, Kansas City, MO, USA or the like) and oxygen consumption will be continuously measured using an open circuit spirometry system (e.g. TrueOne, Parvo Medics, Sandy, UT, USA or similar). Participants will complete a 1-min warm-up at 15 W. The initial work rate will be set to 15 W and gradually increase until the participant reaches volitional fatigue. The work rate will be increased by 10 W per minute or 5 W per minute for participants with mild to moderate (i.e., EDSS of 4.0–5.5) or severe disability (i.e., EDSS of > 6.0), respectively. Participants will be encouraged to maintain a stepping rate of 60–100 steps per minute throughout the test depending on the work rate. Heart rate (Polar FT1 Heart Rate Monitor, Polar Electro Inc., Bethpage, NY, USA), and Ratings of Perceived Exertion (RPE) via the Borg Rating of Perceived Exertion Scale will be recorded every minute. The highest recorded 20-s rate of oxygen consumption value (VO2) will be recorded as peak oxygen consumption (VO2peak), expressed in mL/kg/min, optimally when two or more of the following criteria is satisfied: (1) respiratory exchange ratio (RER) of 1.10 or greater; (2) peak heart rate within 10 beats per minute of age-predicted maximum (i.e., 220-age); or (3) RPE of 17 or greater. The highest recorded power achieved during a 20-s period will be recorded as peak power output (Wpeak). This CPET protocol has previously been used in persons with MS [46], and will be used for measuring changes in aerobic capacity and for prescribing the recumbent stepper exercise training sessions [21].

Walking performance will be assessed by the 6 min walk test (6MWT). Subjects will be instructed to walk at their fastest speed, and to cover as much distance as possible, according to the script of Goldman et al. [47]. Subjects will be notified, without further encouragement, about each expired minute. Distances walked per minute and total distance will be recorded. Subjects walk back and forth along a 30-m hallway turning around cones at each end. In centers without this facility, a square trajectory is allowed given that this has been shown not to compromise results [48].

Cognitive-motor interference during walking will be quantified by a dual task cost (DTC) calculation in the motor and cognitive domains. The DTC calculation is based on a comparison of the performance on a single motor or cognitive task and the motor and cognitive performance during a concurrent motor plus cognitive dual task. The formula is DTC = ((DT-ST)/ST)*100. There are data suggesting that this dual modality testing can give additional insights into the putative benefits of the proposed interventions [49–51]. The single motor task requires the participant to walk at the fastest possible speed while maintaining safety for 60 s. The distance covered is measured. The setting for the 6MWT (30 m corridor) will be used for this test. The single cognitive task entails performing the alternating Latin alphabet for 60 s [52]. During the task, subjects list alternating letters of the alphabet as fast and accurately as possible (i.e., A, C, E, G, etc.). The number of correct letters provided by the subjects is recorded. The test requires working memory and inhibitory control. The cognitive-motor dual task involves walking at fast speed for 60 s with the alternating alphabet test as a concurrent task. Subjects will be instructed to divide attention equally on walking while correctly naming alternating letters of the alphabet.

Physical activity will be determined by accelerometry. Participants will wear the accelerometer (Actigraph; http://actigraphcorp.com/) on an elastic belt around the waist located above the non-dominant hip during the waking hours for 7 days before the first intervention week and during the week following completion of the intervention. This method has proven reliable in people with MS [53]. It will provide data on the degree of lifestyle physical activity (i.e. steps/day and minutes/day of moderate to vigorous physical activity) immediately before and after the intervention phase.

#### Patent reported outcomes (PRO’s)

PRO’s include the HADS, BDI-II, MFIS and PDQ. The MSWS-12, MSIS-29 version 2 and EQ-5D-5L are all standardized self-report outcome measures having strong reliability and validity in people with MS [54, 55] and with evidence supporting their responsiveness in rehabilitation trials [55–57]. Their wide use in MS interventional studies will enable comparisons between studies. The MSWS-12 provides information on the subjective impact of MS on walking and related activities and therefore adds important information regarding what can be obtained from objective measures of walking. Furthermore, it has strong psychometric properties [58]. The MSIS-29 is a disease specific measure of the impact of MS; it has a preference-based tariff [59] for use in sensitivity analyses for the Quality Adjusted Life Year (QALY) outcome, and has been endorsed for use in health economic analyses in MS studies [60]. This will complement the EQ-5D-5L, which is recommended for use in health policy decision making [60]. The FAMS was specifically developed for use with the MS population and has been shown to have adequate reliability and validity within this population. The measure contains 59 questions organized into 6 subscales: mobility, symptoms, emotional well-being, general contentment, thinking/fatigue, and family/social [61]. The thinking/fatigue subscale is composed of 9 items, including questions pertaining to task initiation, task completion, new learning, memory, concentration, and slowness of thought. Participants rate their symptoms on a 5-point Likert type scale. This assessment focuses on the person as a whole, investigating impairments, functional limitations, and disability in many areas of the person’s life. This overview of functional status at all levels is important in determining the impact of cognitive treatment on an individual’s everyday life**.** The FAMS has demonstrated good internal consistency, reliability and validity [61]. The scale has been used successfully to measure change from before to after cognitive rehabilitation [62]. To provide an assessment of anxiety and depression at each assessment, the Hospital Anxiety Depression Scale (HADS) [63] will be administered. The Modified Fatigue Impact Scale (MFIS) [64] will additionally be administered to assess fatigue at each assessment. Subjective cognitive deficits will be assessed with the Perceived Deficits Questionnaire (PDQ) [65].

#### Brain MRI protocol

Brain MRI scans will be obtained using 3.0 Tesla scanners. Budgetary constraints dictate that the MRI is obtained in one third of the sample (i.e. 120 subjects divided equally (*n* = 30) between the four treatment arms). The following sequences will be collected at baseline, termination of the interventions and after 24 weeks of follow up, following a standardized protocol of acquisition and careful guidelines for patients repositioning: axial T2 weighted Turbo Spin Echo (TSE); axial FLAIR; high resolution 3D sagittal T1-weighted sequence; axial DT sequence (55 contiguous, 2.5 mm thick, slices, #DW direction = 64) and T2*-weighted single-shot echo-planar imaging (EPI) during and active cognitive fMRI task and at rest.

For active fMRI, the Go/No-go task will be administered using a block-design, as previously described [66]. Reaction times, omission errors (no response although required), commission errors (false response without adequate cue), and the proportion of correct responses will be recorded using a response-box. Before imaging, participants will be familiarized with the paradigm. The fMRI Go/no-Go paradigm has been used both in cross-sectional [67] and longitudinal [68] studies of people with MS. Notably, a longitudinal (median follow up 20 months) neuropsychological and fMRI evaluation detected significant correlations between worsening of SDMT performances and modification of activation during the Go/no-Go task in several supra- and infratentorial brain regions [68]. Most importantly, the Go/no-Go task has already been validated for multicentric acquisition [66]. During resting state (RS) fMRI, subjects will be instructed to remain motionless, to close their eyes and not to think about anything in particular. Movements will be minimized using foam padding and ear blocks.

The total duration of MRI acquisition (structural plus functional MRI) will be approximately 50 min.

MRI analysis: MRI data acquired for the study will be analyzed centrally at one Neuroimaging Research Unit (Hospital San Raffaele, Milan, Italy).

Lesion and atrophy analysis: Brain T2-hyperintense and T1-hypointense lesion volumes (LV) will be measured on FLAIR and 3D T1-weighted scans, respectively, using a local thresholding segmentation technique (Jim 7.0, Xinapse Systems, West Bergholt, UK). New lesions at follow-up will be counted. Normalized brain (NBV), WM (WMV) and GM (GMV) volumes will be measured on 3D T1-weighted scans using the SIENAx software, after T1-hypointense lesion refilling.^168^ Hippocampal volume will be estimated using FIRST software.

Mapping changes in gray matter (GM) and white matter (WM) structures: Voxel-based Morphometry (VBM) with DARTEL method will be applied to determine between-group differences of GM volumes at baseline, using SPM12 and 3D T1-weighted images. Tensor-based Morphometry (TBM) [69] will be applied to map the longitudinal regional variations of GM volume at the different time points.

Diffusion-weighted images will be corrected for distortions induced by the eddy currents and for head movements, and transformed to MNI (Montreal Neurological Institute) space. Then, using the FMRIB’s Diffusion Toolbox (http://www.fmrib.ox.ac.uk), the DT will be estimated in each voxel by linear regression [70] and mean diffusivity (MD), radial diffusivity (RD), axial diffusivity (AD) and fractional anisotropy (FA) maps derived. Tract-based Spatial Statistics (TBSS) will be used to define the patterns of microstructural WM abnormalities on diffusion tensor images at baseline and their variations during the follow up.

Analysis of fMRI data: Active and RS fMRI data will be pre-processed using SPM12. Activations during the Go/no-Go task will be estimated using SPM12. An independent Component Analysis (ICA) will be used to decompose RS fMRI data into spatially independent maps and time courses, using the GIFT software [71]. Individual functional maps will be converted to Z-scores before entering group statistics, to obtain voxel values comparable across subjects. A systematic process will be applied to inspect and select the components of interest from the estimated ones. The association of each component spatial map with a priori probabilistic maps of GM, WM, and CSF within the MNI space will contributed to identifying the components with a signal change correlated to the GM. Components with a high correlation with cerebrospinal fluid or WM, or with a low correlation with the GM, will be excluded. In addition, to identify components with potentially functional relevance, a frequency analysis of IC time courses will be performed to detect those with a high (50% or greater) spectral power at a low frequency (between 0.01 and 0.05 Hz) [72]. The spatial patterns of the remaining ICs will be sorted out on the basis of their matching with relevant RSNs found in previous studies [73–76] A seed-base RS functional connectivity (RS FC) analysis, using the thalami as a seed, will also be performed to assess modifications of RS FC of the thalamic network in the main study groups and their correlations with clinical scales [77].

#### Standardisation and data quality

To promote data quality, all assessment activites are manualised. Further, before initiating recruitment all PI’s, blinded and unblinded assessors participated in a training session at which all of the tests were discussed and demonstrated. Following this, every site performed a rehearsal incremental CPET on at least one MS patient and sent the data to the responsible PI for review. To ensure that all sites initialise their accelerometers correctly, the assessors themselves wore a device for 7 days, and the data sent to the same PI for review. Similarly, a mock MRI scan will occur at the four centers participating in the MRI substudy. All data will be entered into REDCap [78]. Before entering any data in the actual study database, all assessors will undertake practice lessons and complete a practice certification consisting of a set of ficticious data. Data will be downloaded regularly for quality control purposes and basic reports will be generated via the REDCap system and SAS. Data forms will employ validation and skip pattern logic that provides constrained input whenever possible. Errors or questionable data will be turned into data queries and will be sent by the Data Coordinating Center to the sites for correction and/or clarification. Reports will be produced and include information on data quality, completeness and protocol adherence.

Confidentiality is guaranteed through anonymity. Each participant is given an unique case number, and the Data Coordinating Center at Washington University are not given any identifying data.

#### Power analysis

The primary outcome measure is the SDMT. A four-point improvement on the SDMT is considered clinically useful [37, 38]. Evidence for the reliability of the test was initially obtained in a study of 80 adults administered the test in 2 test sessions approximately 30 days [79]. Comparisons of test scores obtained at times 1 and 2 resulted in a test-retest correlation of 0.80. Individuals tested at baseline obtained a score of 56.79 ± 9.84. Scores increased an average of 3.67 points to 60.46 ± 11.16 at retesting. Such an increase in scores at the second test reflects a “practice effect,” which is commonly seen in test-retest situations [80]. This level of test-retest correlation has been reproduced multiple times and is often higher. We expect a learning curve to be present in all groups and this will in effect wash out in the analyses as our comparisons will utilize comparative changes in means.

Camp et.al. in a primary progressive population studied longitudinally revealed standard deviations of 13.96 at baseline, 15.24 at 1 year and 14.32 at 2 years [81]. Sonder et.al. showed a standard deviation of 14.3. Further, the change over time was generally impacted slightly by the learning curve returning to baseline as the time scale extended [82]. Thus, we can think of changes from baseline to 12 weeks or 6 months in the present study as potentially representing a zero change except for the intervention effect.

The reliability of the SDMT is high, 0.80 or higher as noted above, implying that the correlation between two measures that are not materially changing have high reliability and thus the standard deviation of the change should be relatively small. If we assume that the correlation between two measures is only 0.50, then our estimated standard deviation of the change would be the same as the cross-sectional value (i.e. 10 to 16). However, given the high test-retest correlation, we should assume the correlation between the measures is higher. Assuming it is 0.80, then the estimated standard deviation would be between 6.3 and 10.1 (assuming the cross-sectional standard deviation is 10 or 16 respectively). Thus assuming a standard deviation of the changes of 7 to 8, seems reasonable and 8 may be slightly high, since 16 is at the upper end of most reports. If the standard deviation is 12 cross-sectionally, then the estimated change standard deviation is 7.6. Nevertheless, for sample size estimation it is better to be a bit conservative.

We propose to treat four groups of participants as noted above. We estimated our sample size using a standard 1-factor analysis of variance approach with a Type I error set at 5%. We computed the sample size necessary to achieve 80% power for such a design assuming conservative changes. For simplicity we used 4 points for the combined treatments, *assuming* that we want to demonstrate a clinically meaningful difference on average and that the two interventions are additive; 4 more correct answers has been suggested by the FDA as a meaningful change [37]*.* We also assumed a change of 2 answers for each of the single interventions and 0 for the sham group [83]. (Note *again* that the learning curve and practice effects will contribute equally to each group and the results shown below are the same if we had chosen 6, 4, 4, 2 *or* other combinations with the same relative spread to accommodate the practice effects, etc.)

Table 6 shows that with 90 participants per treatment group at the time of analysis, there is over 80% power to detect differences as specified (4,2,2,0) across the four groups when the standard deviation of the change is 8 points and the overall Type I error to detect any mean differences is 0.05.

**Table 6:** Power for 4 group Analysis of Variance with mean changes in the SDMT of 4 more correct for the combined group; 2 more correct for each of the single intervention groups and 0 change for the Sham Group

**Power**	**n**	**Total N**
0.52751	50.00	200
0.57247	55.00	220
0.61476	60.00	240
0.65423	65.00	260
0.69081	70.00	280
0.72449	75.00	300
0.75533	80.00	320
0.78341	85.00	340
0.80885	90.00	360
0.83179	95.00	380
0.85239	100.00	400

In order to assess the sensitivity of these calculations to assumptions, we conducted a few additional patterns of response and standard deviations. Clearly for any standard deviation smaller than 8, we have more power. For example, if the standard deviation of the change were 7 instead of 8, the power is 91% with 90 patients per group and 81% with 70 per treatment group. If the treatment difference pattern is 4,1,1,0; the power is 86% with four groups assuming a standard deviation of 8.

If the change is less in the combined treatment group, and the pattern is say 3,2,1,0 and a standard deviation of the change is 8, the power is 59%. If the pattern is 3,2,1,0 and the standard deviation of the change is 7, then the power is 71%. Thus, the power is reasonably high for a variety of the patterns. Obviously increasing the sample size in each treatment group increases the power, but we will increase the number recruited and randomized to account for potential dropouts, thus should have ample power under these assumptions.

### Statistical analyses

The statistical analyses will begin with descriptive analyses of baseline characteristics (age, sex, disease duration, EDSS, other physiologic parameters at baseline and over time, medications, etc.), by treatment allocation ((1) EX + CR, (2) EX + CR-S (3) EX-S + CR or (4) EX-S + CR-S). Continuous variables will be summarized using the statistics mean, median, SD, minimum and maximum. Categorical variables will be summarized with frequency counts and percentages. During the trial, drop-outs and losses to follow-up will be compared between groups to ensure high follow-up rates and comparability and that no particular demographic or site is differentially dropping out of one treatment group. The currently supported version of SAS software will be used to perform all data analyses.

Summary tables will indicate the number of subjects with complete data for each measurement, event or outcome. All analyses will be based on available data, unless otherwise stated, and the intent-to-treat principle. Secondary analyses will examine the per protocol analysis population. All confidence intervals will be two-sided and will use 95% confidence levels. Any analyses requiring significance testing will use a two-sided test at the 5% significance level, unless otherwise specified.

Differences in baseline characteristics between groups will be examined in continuous variables, such as age and disease duration using an ANOVA and categorical variables using a Chi-square tests of association. Informative censoring will be examined with potentially biased imputation and non-informative censoring as well as missing-at random assumptions using multiple imputation to provide sensitivity analyses of the primary results.

The primary analysis will utilize an ANOVA and include an interaction term for the combined treatments. A priori contrasts (stated above in the hypothesis section) will be conducted if the overall test of differences amongst the treatment groups achieves statistical significance. For the pairwise comparisons Dunnett’s test will be used to preserve the Type I error rate. Additional analyses will be conducted as sensitivity analyses using Analysis of Covariance (ANCOVA). These will include site, gender, age and other covariates that may be seen to differ amongst the groups at baseline. Multiple imputation will be used to assess the sensitivity of the primary results to dropouts.

Secondary outcomes will be similarly assessed using ANOVA and ANCOVA procedures and, repeated measures mixed models will be used for measurements that are taken between baseline and 12 weeks.

#### Statistical analysis of MRI data

In each group, longitudinal hierarchical linear models will be used to assess changes over time of WM tract DTI measures and average Z-scores of RS functional connectivity (FC), accounting for the repeated measurement design. Statistical analyses of VBM, TBM, MRI active and RS FC maps derived from ICA will be performed using the SPM12 software (whole brain analysis, *p* < 0.05, family-wise error [FWE], corrected for multiple comparisons).

Voxelwise differences of MD, RD, AD and FA values between groups at baseline, and their within-group changes at follow up will be tested using a permutation method (“Randomize” program within FSL) and two-sample and paired t tests, as appropriate (*p* < 0.05 FWE).

Linear regression analysis (using SPM12) will be used to assess the correlations between fMRI activations, RS FC maps and clinical and neuropsychological data.

## Discussion

The present study will be the first combined CR and exercise trial to date in persons with PMS with the potential to change clinical practice; this trial further is the largest combined CR and exercise study in any MS phenotype. As the study is a large international multicenter study, efforts have been made to ensure trial feasibility. Moreover, the collaborating sites have access to patient populations that should allow sufficient recruitment. Efforts have been made to ensure optimal standardization and subsequently best possible data quality. Such efforts relate to 1) comprehensive and detailed assessment and intervention manuals as well as a combination of face-to-face and distance training on how to deliver these, 2) weekly quality control and feedback of the delivered interventions, 3) hotlines in case of questions relating to delivery of the interventions and 4) weekly telephone conferences allowing the widely dispersed centers close communication.

There are well described challenges related to the development of appropriate sham interventions when it comes to exercise trials [84, 85]. Adding to this challenge is the fact that the underlying mechanisms mediating potential exercise induced effects on brain function are poorly understood [86]. A sham-concept that does not significantly improve cardio-respiratory function but still involves attentional and social contact components was therefore chosen as a counterweight to the aerobic exercise intervention [13]. Choosing the appropriate sham treatment is important as it limits the potential of this intervention to alter brain function that is known to accompany aerobic exercise.

The imaging component to our study broadens the scope of our inquiry. MRI techniques are currently being applied to investigate mechanisms related to structural and functional brain plasticity in healthy individuals following training and in neurologically impaired individuals following spontaneous recovery and after rehabilitation interventions. Several authors have used fMRI during active tasks or at rest to evaluate the effects of motor [87, 88] and cognitive [18, 36, 89–92] rehabilitation in MS patients. All of these studies have demonstrated that a modulation of function in brain regions have a crucial role in the trained function which occurs in MS after rehabilitation and is associated with clinical improvement. Whether such changes are possible in people with PMS, is not yet known. Our study therefore has the potential to address the dearth of data in this population and shed light on the degree to which neural plasticity is retained in the context of a progressive disease course.

The impact of cognitive dysfunction in the lives of people with MS is considerable. It is associated with difficulties finding and sustaining employment, maintaining intimate relationships and friendships, pursuing leisure activities and managing basic activities of daily living [93]. Given that we have chosen a primary outcome measure, namely the SDMT, in which a 10% (or 4-point) change over time is known to be clinically significant, should our interventions achieve this threshold, they will acquire the imprimatur of an elusive ecological validity. Moreover, the multinational composition of the research teams all pursuing a shared methodology has the potential to demonstrate that the chosen interventions can transcend language, cultural and indeed institutional barriers that make it difficult to extrapolate results from only a single centre.

The study is expected to conclude by the end of 2022. With a robust sample size that ensures adequate statistical power, the findings, if positive, have the potential to guide clinically meaningful interventions for people with PMS who struggle with all the functional limitations associated with slowed processing speed.

## Data Availability

At completion of the study, only the data coordinating center at Washington University will have access to the final dataset. At conclusion of the study, results will be communicated through individual MS societies from each participating country.

## Abbreviations

ACSM: American College of Sports Medicine
AD: Axial Diffusivity
AHA: American Heart Association
ANART: Adult National Reading Test
ANCOVA: Analysis of Covariance
ANOVA: Analysis of Variance
BDN-II: Beck Depression Inventory – II
BICAMS: Brief International Cognitive Assessment for MS
BVMT-R: Brief Visuospatial Memory Test-Revised
CMI: Cognitive-Motor Interference
CNS: Central Nervous System
CPET: Cardiopulmonary Exercise Test
CR: Cognitive Rehabilitation
CSF: Cerebrospinal Fluid
CVLT: California Verbal Learning Test
CT: Continuous Training
DCT: Dual Task Cost
EDSS: Expanded Disability Status Scale
EPI: Echo-planar Imaging
EQ -5D-5L: European Quality of Life – 5 Dimensions
EX: Exercise
FA: Fractional Anisotropy
FAMS: Functional Assessment of Multiple Sclerosis
fMRI: Functional Magnetic Resonance Imaging
GLTEQ: Godin Leisure-Time Exercise Questionnaire
GM: Grey Matter
GMV: Grey Matter Volume
HADS: Hospital Anxiety and Depression Scale
HIIT: High Intensity Interval Training
HR: Heart Rate
ICA: Independent Component Analysis
IET: Incremental Exercise Test
IQ: Intelligence Quotient
LSD: Lysergic Acid Diethylamide
MD: Mean Diffusivity
MFIS: Modified Fatigue Impact Scale
MNI: Montreal National Institute
MRI: Magnetic Resonance Imaging
MS: Multiple Sclerosis
MSIS-29V2: Multiple Sclerosis Impact Scale – 29 Items Version 2
MSWS-12: Multiple Sclerosis Walking Scale 12 Items
NART: National Adult Reading Test
NBV: Normalized Brain Volume
PCP: Phencyclidine
PDQ-20: Perceived Deficits Questionnaire 20 Items
PI: Principal Investigator
PMS: Progressive Multiple Sclerosis
PPMS: Primary Progressive Multiple Sclerosis
PRO: Patient Reported Outcomes
PS: Processing Speed
QALY: Quality Adjusted Life Year
RD: Radial Diffusivity
RPE: Rate of Perceived Exertion
RRMS: Relapsing Remitting Multiple Sclerosis
RS: Resting State
RSFC: Resting State Functional Connectivity
-S: Sham
SD: Standard Deviation
SDMT: Symbol Digit Modalities Test
SPMS: Secondary Progressive Multiple Sclerosis
TBM: Tensor-based Morphometry
TBSS: Tract-based Spatial Statistics
TSE: Turbo Spin Echo
VBM: Voxel-based Morphometry
W: Watt
WM: White Matter
WMV: White Matter Volume
WR: Work Rate
WTAR: Wechsler Test of Adult Reading
6-MWT: Six Minute Walk Test
